# Sepsis mimics among presumed sepsis patients at intensive care admission: a retrospective observational study

**DOI:** 10.1007/s15010-023-02158-w

**Published:** 2024-01-27

**Authors:** Maria Lengquist, Anjali Varadarajan, Shiva Alestam, Hans Friberg, Attila Frigyesi, Lisa Mellhammar

**Affiliations:** 1 Department of Clinical Medicine, Lund University, Lund, Sweden; 2 Department of Anaesthesia and Intensive Care, Skåne University Hospital, Lund, Sweden; 3 Department of Anaesthesia and Intensive Care, Skåne University Hospital, Malmö, Sweden; 4 Department of Infectious Diseases, Skåne University Hospital, Lund, Sweden

**Keywords:** Sepsis, Critical care, Infections, Biomarkers, C-reactive protein, Leukocytes, Shock, Septic

## Abstract

**Background:**

Diagnosing sepsis remains a challenge because of the lack of gold-standard diagnostics. Since there are no simple, broadly accepted criteria for infection, there is a risk of misclassifying sepsis patients (sepsis mimics) among patients with organ failure. The main objective of this study was to investigate the proportion of non-infected patients (sepsis mimics) in ICU patients with presumed sepsis at intensive care unit (ICU) admission.

**Methods:**

Adult patients were screened retrospectively during 3.5 years in four ICUs in Sweden for fulfilment of the sepsis-3 criteria at ICU admission (presumed sepsis). Proxy criteria for suspected infection were sampled blood culture(s) and concomitant antibiotic administration. Culture-negative presumed sepsis patients were screened for infection according to the Linder-Mellhammar Criteria of Infection (LMCI). Sepsis mimics were defined as without probable infection according to the LMCI. Confirmed sepsis was defined as presumed sepsis after the exclusion of sepsis mimics.

**Results:**

In the ICU presumed sepsis cohort (2664 patients), 25% were considered sepsis mimics. The most common reasons for ICU admission among sepsis mimics were acute heart failure and unspecific respiratory failure. Comparing sepsis mimics and confirmed sepsis showed that confirmed sepsis patients were slightly more severely ill but had similar mortality. C-reactive protein had modest discriminatory power (AUROC 0.71) with confirmed sepsis as the outcome.

**Conclusions:**

One-fourth of a presumed ICU sepsis population identified with the sepsis-3 criteria could be considered sepsis mimics. The high proportion of sepsis mimics has a potential dilutional effect on the presumed sepsis population, which threatens the validity of results from sepsis studies using recommended sepsis criteria.

**Supplementary Information:**

The online version contains supplementary material available at 10.1007/s15010-023-02158-w.

## Background

Sepsis is a frequent cause of Intensive Care Unit (ICU) admission, but criteria for sepsis identification vary between studies. The choice of criteria determines what cohorts are included and can affect treatment effects in intervention studies. [1, 2]. According to the sepsis-3 task force, the suggested clinical sepsis criteria are suspected or verified infection in combination with organ failure measured as an increase in Sequential Organ Failure Assessment (SOFA) score with two points or more [3]. Organ failure at ICU admission is common [4]. Therefore, the fulfilment of sepsis criteria will probably be determined by infection criteria fulfilment for a majority of the ICU patients.

There are no gold standard infection criteria, and the choice of infection criteria is probably the major cause for the difference in sepsis inclusion between studies. The sepsis-3 task force has suggested microbiological culture sampling in combination with antibiotic administration as criteria for suspected infection to be used for research purposes [3]. Physician suspicion of infection is another common approach [5, 6]. These proxy criteria for infection are easy to apply in large datasets and can be used in prospective studies. Other criteria for infection exist, such as the International Sepsis Forum definitions of infection in the ICU by Calandra et al. or the Center for Disease Control and Prevention (CDC) criteria[7, 8]. These criteria require a more in-depth assessment of clinical data, often rely on microbiological test results, and are consequently unsuitable for prospective studies. A new tool for diagnosing infection was published in 2022, the Linder-Mellhammar Criteria of Infection (LMCI). The aim was for a more comprehensive yet simple set of criteria to diagnose infection in 13 potential foci (compared to 6 in Calandra et al.). The LMCI classifies a patient as infected with four degrees of certainty: no, possible, probable or proven infection. The LMCI includes but does not rely on microbiological test results and is intended to be used in the ICU and hospital wards. A comparison with Calandra et al. showed higher agreement with expert adjudication for LMCI[9].

Broadly applicable criteria, such as culture sampling and antibiotic administration or clinical suspicion, risk including false positive patients, so-called sepsis mimics, in an ICU setting. This is because the threshold to suspect and treat an infection is low in critical illness. The issue of sepsis mimics was highlighted in the last version of the Surviving Sepsis Campaign [10]. The proportion of sepsis mimics has only been analysed in a few ICU studies, none using the sepsis-3 criteria, but all using physician suspicion of infection as a proxy criterion. Contou et al. investigated 508 patients with suspected infection needing vasopressor therapy at ICU admission and found that 11% were sepsis mimics [11]. Heffner et al. investigated 211 patients who were admitted to the ICU from the emergency department with sepsis-2 criteria for septic shock and found 16% to be sepsis mimics [12]. Klouwenberg et al. investigated 2579 patients who fulfilled sepsis-2-criteria at ICU admission and found that 13% had no infection [13].

### Objectives

The objectives of this study were to investigate

- The proportion of sepsis mimics among patients with presumed sepsis according to sepsis-3 criteria, including recommended proxy criteria for infection, at ICU admission
- How these sepsis mimics differ from confirmed sepsis patients
- The ability of frequently used clinical infection tools to discriminate between confirmed sepsis and sepsis mimics/non-sepsis

## Methods

### Study design

This study was a retrospective observational study with data collected mainly through electronic medical record (EMR) review. The Strengthening the Reporting of Observational Studies in Epidemiology (STROBE) guidelines were followed[14].

### Setting

Patients in this study were admitted to one of four general ICUs at Skåne University Hospital in Lund and Malmö, Helsingborg Hospital and Kristianstad Hospital, all located in the south of Sweden. The period of patient inclusion was from 2015 (different starting dates depending on site) until December 31, 2018. This period reflected the Swecrit biobank’s collection period, of which this study is an adjunct[15]. The study size was a convenience sample of all ICU admissions during a fixed period.

### Participants

All adult ICU patients ($\ge$18 years old) were retrospectively screened for the fulfilment of sepsis-3 criteria around the time of ICU admission (see previous publication for details [16]). Sepsis was defined as SOFA-score$\ge$2 within the time frame 1 h before to 1 h after ICU admission in combination with suspected infection. Proxy criteria for suspected infection were blood cultures sampled within 24 h before to 24 h after ICU admission, and $\ge$1 dose of antibiotic therapy administered, according to recommendations of the sepsis-3 task force [3]. The exclusion criteria were direct transfer from another ICU or elective ICU admission after surgery. All patients included in the analyses are termed *presumed sepsis*.

Culture-negative presumed sepsis patients were screened for infection using the LMCI[9]. The cutoff for infection was chosen at 3 points or more within at least one focus of infection (probable or proven infection) or fulfilment of criteria for possible or definite endocarditis according to the modified Duke criteria[17]. Failure to reach this cutoff within any focus led to the classification of a *sepsis mimic*. The remaining sepsis patients, after the exclusion of sepsis mimics, were called *confirmed sepsis*. Sepsis mimics were used as a reference group to confirmed sepsis. In summary, this study’s gold standard for infection was culture positivity, or probable infection, according to LMCI for culture-negative patients.

### Data sources

The LMCI review of EMR was performed by three research group members, two intensive care residents and one intensive care attending. Uncertainties were discussed with co-author LM, who is an infectious medicine specialist. A review of the comorbidities in the EMR was performed by a group of trained data collectors, mainly intensive care residents and medical students.

Survival data, diagnostic coding, Simplified Acute Physiology Score-3 (SAPS-3) and SOFA scores, vital signs and variables used to calculate SAPS-3 and SOFA were based on data entered by the treating physician into the PasIVA software, used to collect data for the Swedish Intensive Care Registry (SIR). Laboratory values and microbiological test results were automatically extracted from the hospital laboratory electronic system. See Supplement 3 for the data sources of variables used in Table 1.

### Variables

Culture-negative presumed sepsis was defined as without clinically relevant pathogen detected in any sample within 48 h before to 48 h after ICU admission. See Supplement 1 for the definition of clinically relevant culture results.

Sepsis mimics were assessed for retrospective causes of ICU admission to identify what diagnoses constitute sepsis mimics in the ICU. All medical information available at the EMR review was considered, including relevant medical investigations performed after ICU admission and autopsies. The diagnoses were applied in an inductive manner, where the medical state assessed to have primarily contributed to ICU admission was chosen as a diagnosis at ICU admission. Diagnoses were aggregated into diagnostic categories to facilitate reporting. A combined pie/doughnut chart was constructed based on the diagnostic categories, with diagnoses <1.5% of the sepsis mimics subgroup not being labelled. All levels of diagnoses/categories, descriptions of all diagnoses and frequency counts were reported separately in Supplement 2. Two mock patient case reports were constructed based on an aggregation of multiple patients and with details distorted to ensure patient confidentiality.

A physician’s suspicion of infection was defined as an explicit suspicion of infection or sepsis by any physician during ICU admission, as stated in the EMR.

Shock was defined as using vasopressor (norepinephrine or epinephrine) at ICU admission, combined with a lactate level of >2 mmol/L.

Sepsis diagnosis was defined as ICD-10 codes R57.2, R65.1, and A41.9 as a main or secondary diagnosis at ICU discharge.

### Quantitative variables

For the biomarkers C-reactive protein (CRP), procalcitonin (PCT), white blood cell count (WBC) and lactate, the highest values within the time frame 48 h before to 48 h after ICU admission were identified. The biomarkers that were used in diagnostic testing were dichotomised according to receiver operating characteristics curve (ROC)-derived optimal cutoffs (Youden’s index)[18].

Body temperature was the highest recorded within 1 h before to 1 h after ICU admission. Fever was defined as temperature $> 38^{\circ } \hbox {C}$ and hypothermia as $< 36^{\circ } \hbox {C}$.

See Supplement 3 for an exhaustive list of variable definitions and data sources for Table 1.

### Statistical methods

All data handling procedures and statistical analyses were performed using RStudio. Median values and interquartile ranges (IQRs) were reported for continuous variables. Despite SOFA scores being an ordinal variable, mean value and standard deviation (SD) were reported for SOFA scores since we believed this better illustrated the distribution than a presentation of frequencies of all SOFA-score levels.

To assess for a difference in the location of two independent variables, the Wilcoxon rank-sum test (Mann–Whitney *U* test) was used. Differences in proportions were assessed using Pearson’s $\chi ^2$ test. For all hypotheses testing, *p*-values <0.05 were considered significant.

Commonly used variables in the clinical setting (CRP, PCT, WBC and body temperature) were assessed in ROC analyses and diagnostic testing, with dichotomised variables and values above the cutoff considered a positive test to identify confirmed sepsis (positive outcome).

If data were missing in hypothesis testing, ROC analyses or diagnostic testing, the patient was excluded from analyses using that specific variable.

Four sensitivity analyses were performed. We assessed the change in the proportion of sepsis mimics when only patients (1) fulfilling shock criteria were included and (2) with $\ge$4 days of antibiotic therapy among patients with available data on antibiotic therapy. Additional sensitivity analyses were performed with (3) stricter criteria and (4) more liberal criteria for infection according to LMCI:

(3) The cutoff was elevated to $\ge$4 points (proven infection) for any focus. The modified Duke criteria were changed to only include definite endocarditis. (4) The cutoff was lowered to $\ge$2 points (possible infection) for any focus, except for the gastrointestinal (GI) focus, since leukocytosis or fever is enough to give 2 points there. The modified Duke criteria for endocarditis were not changed.

See Supplement 1 for details on the method for sensitivity analysis two.

### Bias

The LMCI has some subjectivity (e.g. interpretation of radiological results). To minimise differences in assessment between the data collectors, uncertainties were discussed and jointly agreed on between the three investigators and the infectious disease specialist.

## Results

### Participants

**Fig. 1:**
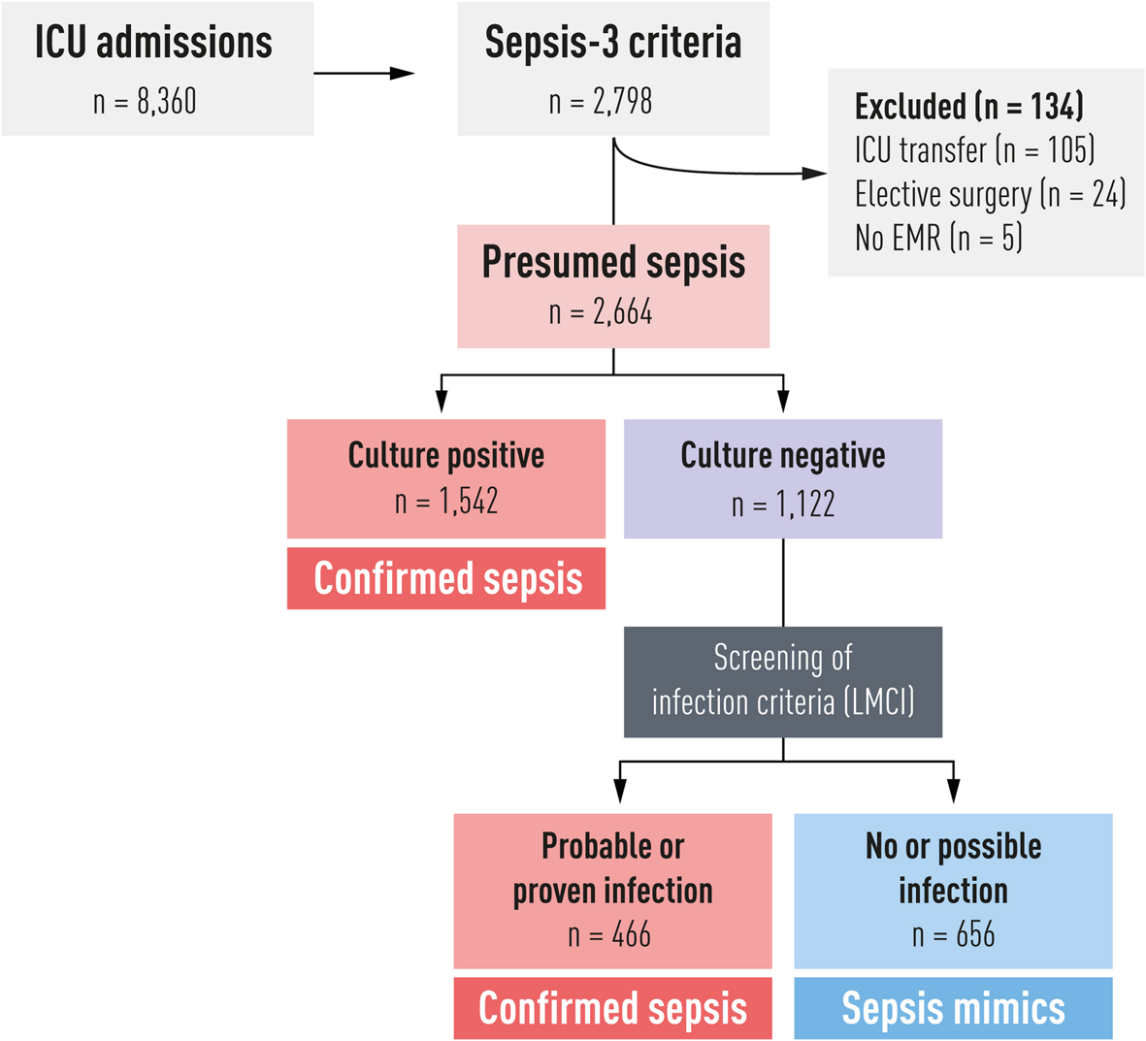
Flow chart of sepsis inclusion and exclusion. Sepsis-3 criteria fulfilment was defined as SOFA-score$\ge$2 and suspected infection at ICU admission. Proxy criteria for suspected infection were blood cultures sampled and antibiotic therapy administered. Culture negativity was defined as no clinically relevant cultures within 48 h before to 48 h after ICU admission. The Linder-Mellhammar criteria of infection (LMCI) is an infection classification tool published in 2022, which classifies a patient as infected with four degrees of certainty: no, possible, probable or proven infection. The patients who had presumed sepsis but were culture negative and had no or possible infection according to LMCI were labelled as *sepsis mimics*. After excluding sepsis mimics, the remaining presumed sepsis patients were labelled *confirmed sepsis*. *ICU* intensive care unit, *EMR* Electronic medical record, *LMCI* Linder-Mellhammar criteria of infection

Of 8360 ICU admissions during the study period, 2664 (32%) fulfilled the operational sepsis-3 criteria after exclusion criteria and were labelled presumed sepsis. Of these, 1122 were culture-negative and subject to review according to the LMCI. See flow chart Fig. 1.

### Main results

#### Proportion of sepsis mimics

Out of the 2664 ICU patients with presumed sepsis, 656 (25%, 95% confidence interval 23–26%) were culture negative and did not fulfil criteria for probable infection according to the LMCI and were thus labelled sepsis mimics.

#### Sepsis mimics diagnoses

**Fig. 2:**
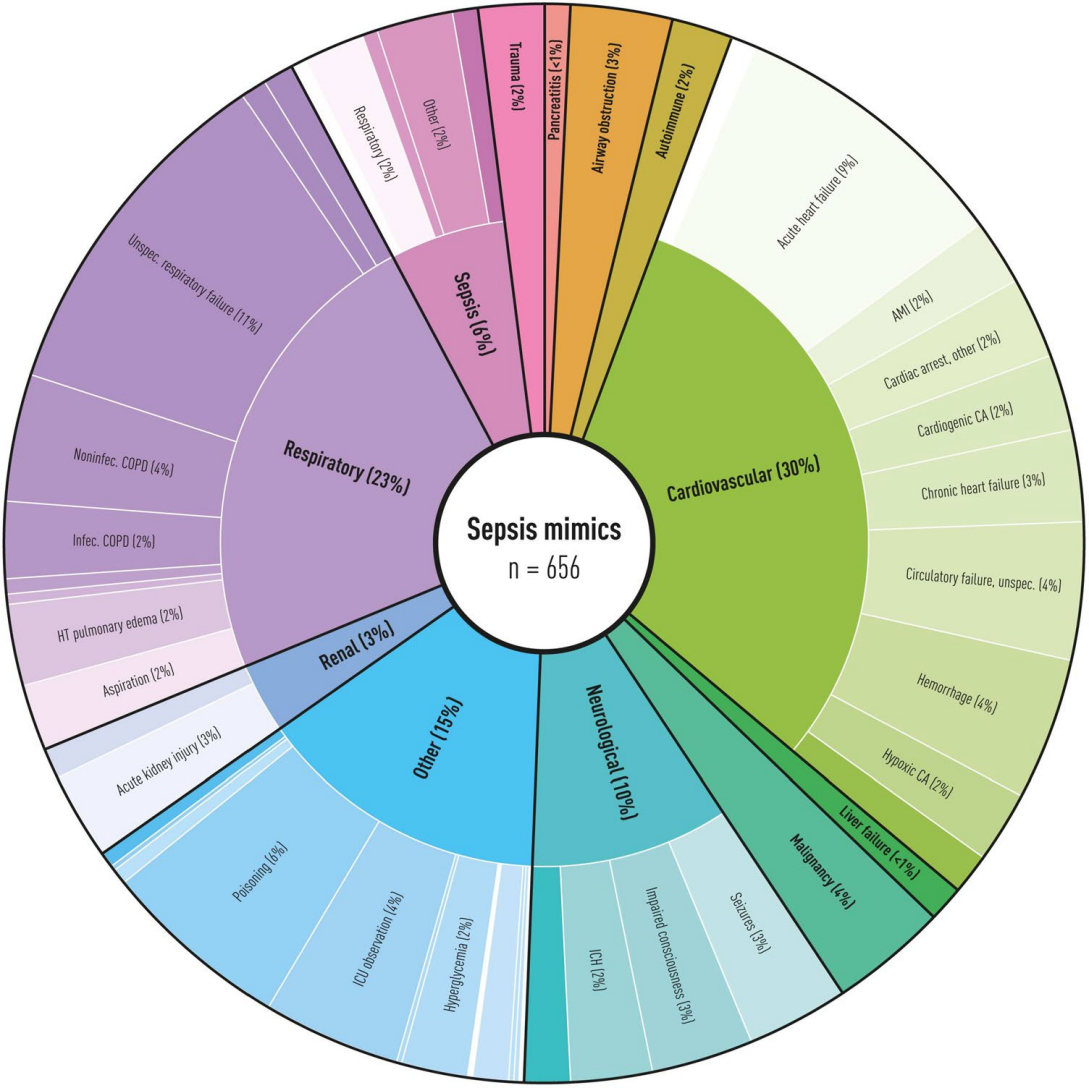
Sepsis mimics diagnoses. The chart illustrates diagnoses (outer circle) and diagnostic categories (inner circle) among sepsis mimics. Diagnoses with less than 1.5% of patients are not presented in the chart. The exact number of patients with each diagnosis can be found in Supplement 2. *AMI* acute myocardial infarction, *CA* cardiac arrest, *ICH* intracranial haemorrhage, *ICU* intensive care unit, *HT* hypertensive, *COPD* chronic obstructive pulmonary disease

Most sepsis mimics had cardiovascular or respiratory diagnoses as causes for ICU admissions, such as unspecific respiratory failure, acute heart failure or cardiac arrest. See Fig. 2 for an illustration of the diagnoses in sepsis mimics. See Supplement 2 for definitions of diagnoses and diagnostic categories in sepsis mimics and frequency counts. Two illustrative patient cases from the most common categories are presented in text box 1.

Text box 1: mock case reports of sepsis mimics **Respiratory failure - COPD exacerbation.**

Sixty-year-old male, chronic smoker. Dyspnea, wheezing and unconsciousness in the emergency room. Afebrile. Distant crepitations over the right hemithorax on auscultation. Hypoxic and hypercapnic (PaCO_2_ 13 kPa). CRP 28 mg/L, WBC $11\hbox { x }10^{9}$/L, lactate 2 mmol/L. The working diagnosis was pneumonia. A chest x-ray showed pleural effusions but no pulmonary infiltrates. Treatment was given with antibiotics, corticosteroids, inhalation of bronchodilators, and invasive ventilation. There was a rapid improvement and subsequent discharge from the ICU.

**Circulatory failure - acute heart failure.**

Seventy-eight-year-old ex-smoking female with diabetes mellitus and hypertension. Presents to the emergency department with abdominal pain, hypoxia, mottled skin, hypotension, and hypothermia. The working diagnosis was abdominal sepsis. Treatment was given with antibiotics, fluids, norepinephrine, and invasive ventilation. CT thorax and abdomen showed pulmonary oedema and a swollen liver. CRP 50 mg/L, WBC 16x10^9^/L, lactate 5 mmol/L. Echocardiography showed severe biventricular failure. A rapid deterioration led to circulatory collapse and death in the ICU.

#### Differences between sepsis mimics and confirmed sepsis

The sepsis mimics subgroup differed in some aspects from the confirmed sepsis population (see Table 1). In sepsis mimics, the bedside physicians had less suspicion of infection (70% vs 87%, p<0.05). There was no difference in hospital length of stay before ICU admission. The disease severity was higher in the confirmed sepsis group, as measured with the SAPS-3 and SOFA scores. The confirmed sepsis group had a higher need for vasopressors, but lactate levels were similar. The confirmed sepsis group had a higher degree of respiratory failure according to the oxygenation index (PF-ratio), as well as the subsequent need for invasive ventilation. Among the different organ-specific SOFA scores, only the neurological score was higher in sepsis mimics. The other organ-specific SOFA scores were higher in confirmed sepsis. The proportion with pre-ICU comorbidities only differed regarding immunosuppression (more common in the confirmed sepsis group) and cardiovascular and respiratory disease (more common in the mimics group), although the differences were small.

#### Biomarker and body temperature differences

Among the biomarkers, CRP and PCT values were markedly lower in sepsis mimics than in the confirmed sepsis group. The absolute difference was small in WBC between sepsis mimics and confirmed sepsis. Fever was more common in the confirmed sepsis group, and hypothermia was less common. See Fig. 4.

### Outcome data

Despite the sepsis mimics group being less severely ill at ICU admission, the crude mortality did not differ between the mimics and confirmed sepsis. The ICU and hospital stay was longer, and the need for continuous renal replacement therapy (CRRT) and invasive ventilation during the ICU stay was higher in the confirmed sepsis group. See Table 1.

### Other analyses

#### ROC analysis and diagnostic testing of biomarkers and body temperature

The ability of CRP, PCT, WBC and body temperature to discriminate between sepsis mimics and confirmed sepsis was tested using ROC analysis. CRP and PCT had AUCs around 0.7, but WBC and temperature had low discriminatory capabilities, with AUCs closer to 0.5.

High values of dichotomised CRP, PCT, WBC and temperature (positive test) were tested against confirmed sepsis (positive outcome) among presumed sepsis patients. Discriminatory capabilities were modest for all four tests, with CRP, WBC and temperature yielding specificities around 80% but with sensitivities between 25–51%. Positive predictive values (PPV) were high for all four tests, owing to the high prevalence of the outcome (confirmed sepsis) in the presumed sepsis group. See Table 2.

ROC analyses and diagnostic testing were also performed in the whole ICU population (see Table S1 in Supplement 1), with confirmed sepsis (n=2008) still being the positive outcome, but the reference group being all non-sepsis (including sepsis mimics), n=6352. AUCs improved for all four tests, with lower optimal cutoffs for CRP and WBC. The dichotomised tests yielded sensitivities and specificities that were more similar (between 47–70%), except for temperature, which had much higher specificity (91%) than sensitivity (25%).

#### Sensitivity analyses - proportion sepsis mimics with altered criteria for sepsis and mimics

Sensitivity analyses were performed to assess the proportion of sepsis mimics by altering the criteria for presumed sepsis and sepsis mimics. See Fig. 5.

**Fig. 3:**
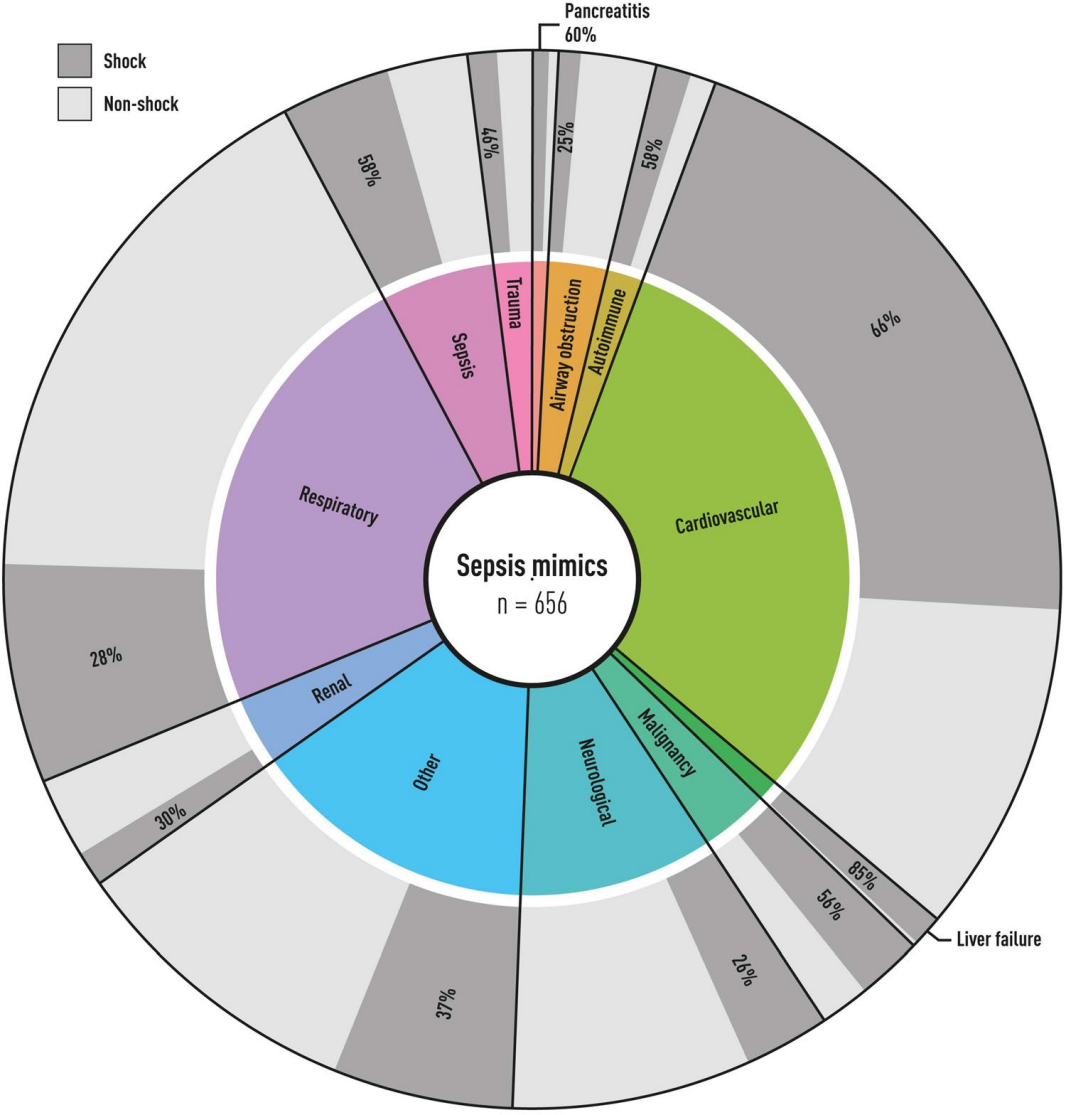
Proportion of patients with shock within each diagnostic category. The outer circle of the chart illustrates the proportion of patients within each diagnostic category fulfilling shock criteria (vasopressor use and lactate>2 at ICU admission). The proportion with shock was highly variable, ranging from 30% in the renal category to 85% in liver failure. Subsequently, the pattern of diagnoses would be different if only patients who fulfilled shock criteria were included

If only presumed sepsis patients with shock (vasopressor and lactate>2) were included, the proportion of sepsis mimics fell to 21%. The pattern of diagnoses would also be different if only patients with shock were included, as illustrated in Fig. 3.

Changing the proxy criteria for infection to only include patients with $\ge$4 days of antibiotic treatment, the number with presumed sepsis was reduced, and the proportion of sepsis mimics was 20% (see Figure 1 in Supplement 1).

Using other cutoffs for LMCI, the proportion of sepsis mimics was 29% with the stricter infection criteria ($\ge$4 LMCI points, proven infection) and 14% with the more liberal infection criteria ($\ge$2 LMCI points, at least possible infection).

The change in the proportion of sepsis mimics was statistically significant (p-value<0.05) for all sensitivity analyses (see Table S2 in Supplement 1).

## Discussion

### Key results

In this retrospective observational study, we found that 25% of the patients with presumed sepsis at ICU admission did not fulfil more accurate infection criteria and could, therefore, be considered sepsis mimics. CRP, PCT, WBC and temperature were found to have low to moderate discriminatory power in distinguishing confirmed sepsis from sepsis mimics.

The sepsis mimics group was heterogeneous, with many non-infectious reasons for ICU admission. However, infection was initially suspected in most sepsis mimics (70%), reflecting the low threshold of suspecting infection in ICU patients. Most had non-infectious circulatory or respiratory causes for ICU admission, such as acute heart failure or mixed or unspecified respiratory failure. A small proportion (6%) of the sepsis mimics found using the LCMI criteria were considered to have sepsis in a clinical review. Of these sepsis mimics, most had sepsis of other/mixed origin or respiratory tract infection. The reason for them not fulfilling the LMCI criteria for probable infection could be a rapid clinical course not allowing for radiology to be performed or a respiratory tract infection not presenting with infectious infiltrate on radiology. This illustrates the discrepancy between what is clinically considered sepsis and the criteria used for research.

The confirmed sepsis group had a higher degree of shock due to a higher proportion of vasopressor need, as the lactate levels were similar. In sepsis mimics, lactatemia was probably caused by reasons other than sustained hypoperfusion.

Compared to previous ICU studies of sepsis mimics using physician suspicion of infection as proxy criteria for infection, we found almost twice the proportion of sepsis mimics (11–18% vs 25%) [11–13]. This is partly due to our study including all patients with presumed sepsis instead of only including presumed septic shock patients, highlighted by the slight decrease in sepsis mimics to 21% if only presumed shock patients were included. This fraction is still higher than found in previous studies, probably due to the choice of infection proxy criteria, with blood culture sampling and antibiotic therapy yielding more sepsis mimics than physician suspicion in an ICU setting. The cutoff for infection and choice of infection classification tool also matters: if Klouwenberg et al. had set their infection cutoff to probable or proven infection (not possible), their proportion of sepsis mimics would have been 43% instead of 13%. Our sensitivity analysis also illustrated this, which yielded highly variable proportions of sepsis mimics depending on the LMCI cutoff. Klouwenberg et al. used Calandra and CDC infection criteria, which only have a 66% overlap with the LMCI in ICU patients, which complicates comparisons[7–9]. The diagnoses that constitute sepsis mimics differed as well, with our most frequent mimic categories not being found in Contou et al., who instead reported adverse drug reactions, vascular disease and malignancies as the most frequent mimic categories. Only including shock patients changed our diagnoses, omitting a majority of the sepsis mimics with respiratory failure.

Using diagnostic coding to identify sepsis will also include possible sepsis mimics, as 14% of our cohort’s sepsis mimics had an ICD-10 sepsis diagnosis registered. Diagnostic coding will also underestimate criteria-based sepsis, leaving diagnostic coding unsuitable for sepsis identification[16].

The variation in sepsis inclusion, depending on slight changes in criteria, illustrates the methodological difficulty in sepsis research, and there is no clear answer to which infection proxy criteria should be used for criteria-based sepsis research. Noteworthy, however, is that all our sensitivity analyses yielded a high and non-negligible proportion of sepsis mimics. Different criteria will probably be suitable depending on the study design, but using only a limited selection of clearly defined criteria will facilitate generalisability and comparison between studies. Prospective studies cannot rely on microbiological test results, which are present after a few days. Still, more detailed clinical criteria (such as the LMCI, including microbiological test results) can be used for retrospective sensitivity analyses of prospective studies to assess the robustness of the results.

Large datasets derived from registries will probably have to rely on simple proxy criteria for infection. Still, the high risk of sepsis mimics inclusion needs to be considered. The trend towards using big data and machine learning algorithms makes the issue of sepsis mimics highly important. However, emerging methods to automatically collect clinical data from EMR could lead to more simplified screening of complex infection classification tools, hopefully improving sepsis identification.

The investigation of CRP, PCT, WBC and body temperature for diagnostic purposes with sepsis mimics as the control group has relevance to the research methodology. None of these clinical markers has high enough discriminatory power to be used in adjunct to the simple infection proxy criteria to identify sepsis according to more detailed criteria (LMCI) and single out sepsis mimics. The +LR of WBC close to 1 means that the test adds little to the assessment of sepsis, which is in line with previous research concluding the uselessness of WBC in sepsis[19]. An ideal marker as an adjunct to infection proxy criteria would have a high specificity to minimise the inclusion of sepsis mimics, which was the case for CRP, WBC and temperature. The low sensitivity would, however, leave many with confirmed sepsis to not be included in a final cohort.

These markers also have potential clinical relevance. The ability to discriminate between confirmed sepsis and all non-sepsis in the ICU was modest. CRP and PCT had better discriminatory power than WBC and temperature. Neither had a high sensitivity to be acceptable as decision support to withhold antibiotics, which is well established[20]. The optimal cutoff for CRP, 124 mg/L, was above the range of previously suggested cutoffs of 12–90 mg/L ([21]. Conclusions regarding PCT are difficult to draw because of the large proportion of missing PCT values and the high likelihood of selection bias in PCT differences in our study.

### Limitations

In this study, screening for the fulfilment of LMCI and physician suspicion of infection was not performed on the entire ICU population. There might be patients with SOFA$\ge$ 2 who fulfilled infection criteria according to LMCI at admission but did not have blood cultures sampled and were not included as presumed sepsis. If the LMCI were screened in the whole ICU population, it would have been possible to test the ability of the infection proxy criteria (blood culture sampling and antibiotic treatment) and the physician’s suspicion of infection as a test to identify sepsis correctly with LMCI as the gold standard for infection/sepsis.

Infection criteria tools, such as LMCI, have limitations since radiology, microbiological samples and other clinical workups are not performed in every patient due to low suspicion of positive findings or rapid clinical deterioration of the patient, which could be a source of selection bias. Since more severely ill patients are probably more thoroughly investigated, the proportion of confirmed sepsis could be higher among the more severely ill, contributing to the differences in SAPS-3 and SOFA scores between confirmed sepsis and sepsis mimics.

We did not investigate interobserver variability between investigators. We can, therefore, not rule out that the results would have differed slightly if the review of all cases was done independently by all three investigators and a result was jointly agreed on. In the original publication of the LMCI, an interobserver agreement of 77% was reported, and if looking at only those re-classified from non-infected to infected, that proportion was 12%.

The high proportion of missing PCT values is probably due to selection bias since PCT was not tested routinely in the included ICUs at the time of the study. Physicians ordered PCT on clinical indication, and the proportion of available PCT was higher in the confirmed sepsis group, i.e. not missing at random.

We analysed only single biomarker values in this study. If biomarker trajectories had been analysed instead, these biomarkers could have better clinical performance [20].

The effort of trying to categorise patients with critical illness into clinical syndromes, such as sepsis, is under investigation since the heterogeneity in pathophysiology is not taken into account [22]. The current sepsis criteria might become obsolete in the near future, but as long as they are in use, their weaknesses must be evaluated.

### Interpretation

The poor ability of frequently used clinical tests to identify confirmed sepsis in a presumed sepsis cohort prompts the need for more precise biomarkers or combinations of biomarkers for diagnostic purposes.

The LMCI criteria are labour-intensive, and a future research topic could be to find simpler proxy criteria that can identify infection and sepsis more precisely.

### Generalisability

The high proportion of sepsis mimics among presumed sepsis in our study has relevance as studies using the same criteria for presumed sepsis in an ICU setting similar to ours can expect a similarly high proportion of sepsis mimics. Using the infection proxy criteria of blood culture sampling and antibiotic administration can also be a source of selection bias, especially between ICU and non-ICU settings since the threshold to obtain blood cultures and administer antibiotics probably is lower in critically ill ICU patients. Using this infection proxy criterion can at first glance seem like an objective criterion, but the threshold to obtain blood cultures and administer antibiotics can be highly variable between physicians, wards and hospitals.

## Conclusions

One-fourth of a presumed sepsis population identified with the sepsis-3 criteria could be considered sepsis mimics. The ability of frequently used clinical markers of infection to improve sepsis identification was low to modest. The high proportion of sepsis mimics has a potential dilutional effect on the presumed sepsis population, which threatens the validity of results from sepsis studies using recommended sepsis criteria.

**Table 1:** Descriptive statistics comparing sepsis mimics and the confirmed sepsis population

	Sepsis mimics	Confirmed sepsis	*P* value
n	656	2008	
Age, years (median [IQR])	69 [60-76]	69 [59-76]	0.33
Male sex (%)	403 (61)	1163 (58)	0.12

Status at ICU admission			
Physician suspicion of infection (%)	*459 (70)*	*1727 (86)*	<*0.001*
Antibiotics before blood culture (%)	238 (36)	663 (33)	0.14
Intra-hospital admittance to ICU (%)	432 (66)	1305 (65)	0.72
Hospital LOS before ICU if >0, days (median [IQR])	4 [2-9]	4 [2-11]	0.75
Hospital LOS before ICU >48h (%)	154 (24)	441 (22)	0.45

Biomarkers at ICU admission			
Lactate, mmol/L (median [IQR])	3.9 [2.2-7.0]	3.7 [2.3-6.7]	0.65
C-reactive protein, mg/L (median [IQR])	*92 [30-190]*	*215 [99-319]*	<*0.001*
Procalcitonin, mcg/L (median [IQR])	*1.1 [0.3-6.1]*	*7.3 [1.2-43]*	<*0.001*
WBC, $10^{9}$/L (median [IQR])	*16.2 [11.9-20.9]*	*16.8 [11.8-23.7]*	*0.032*

Physiology at ICU admission			
Shock (%)	*300 (46)*	*1136 (57)*	<*0.001*
Vasopressor use (%)	*352 (54)*	*1301 (65)*	<*0.001*
PF-ratio, kPa (median [IQR])	*27 [18-42]*	*23 [15-37]*	<*0.001*
Body temperature, $^{\circ }\hbox {C}$ (median [IQR])	*37.0 [36.1-37.6]*	*37.2 [36.5-38.1]*	<*0.001*
Body temperature $> { 38}^{\circ }\hbox {C}$ (%)	*101 (16)*	*507 (25)*	<*0.001*
Hypothermia (%)	*120 (18)*	*261 (13)*	*0.001*

Diagnostic coding			
Sepsis ICD-10 diagnosis (%)	*91 (14)*	*949 (47)*	<*0.001*

SAPS-3 and SOFA at ICU admission			
SAPS-3 score (median [IQR])	*64 [56-74]*	*67 [57-77]*	<*0.001*
SOFA score (mean [SD])	*7.4 (3.5)*	*8.2 (3.6)*	<*0.001*
Respiratory SOFA (mean [SD])	*2.2 (1.3)*	*2.5 (1.2)*	<*0.001*
Coagulation SOFA (mean [SD])	*0.4 (0.8)*	*0.5 (0.9)*	*0.003*
Liver SOFA (mean [SD])	*0.4 (0.8)*	*0.4 (0.8)*	*0.03*
Cardiovascular SOFA (mean [SD])	*1.9 (1.7)*	*2.4 (1.7)*	<*0.001*
Neurological SOFA (mean [SD])	*1.6 (1.5)*	*1.3 (1.4)*	<*0.001*
Renal SOFA (mean [SD])	*1.3 (1.5)*	*1.5 (1.6)*	*0.03*

Comorbidities			
Immunosuppression (%)	*100 (15)*	*391 (20)*	*0.02*
Diabetes mellitus (%)	167 (26)	486 (24)	0.55
Cardiovascular disease (%)	*431 (66)*	*1126 (56)*	<*0.001*
Respiratory disease (%)	*181 (28)*	*432 (22)*	*0.002*
Liver disease (%)	39 (5.9)	111 (5.5)	0.76
Malignancy (%)	80 (12)	252 (13)	0.86
Renal disease (%)	84 (13)	216 (11)	0.17
Haematological disease (%)	43 (6.6)	133 (6.6)	1.0
Body Mass Index, kg/m^2^ (median [IQR])	27 [23-31]	27 [23-31]	0.18

Outcomes			
ICU LOS, days (median [IQR])	*1.8 [0.8-3.3]*	*2.6 [1.1-5.2]*	<*0.001*
Hospital LOS, days (median [IQR])	*11 [5-23]*	*14 [7-29]*	<*0.001*
ICU mortality (%)	90 (14)	319 (16)	0.20
Hospital mortality (%)	185 (28)	614 (31)	0.27
30-day mortality (%)	172 (27)	573 (29)	0.29
CRRT during ICU stay (%)	*73 (11)*	*341 (17)*	<*0.001*
Invasive ventilation during ICU stay (%)	*344 (52)*	*1167 (58)*	*0.012*

Variables in italics have a p-value <0.05 derived from hypothesis testing

*ICU* intensive care unit, *LOS* length of stay, *CRP* C-reactive protein, *PCT* procalcitonin. *WBC* white blood cell count, *PF-ratio* PaO_2_/FiO_2_-ratio (oxygenation index), *SAPS-3* simplified acute physiology score-3, *SOFA* sequential organ failure assessment, *CRRT* continuous renal replacement therapy

**Table 2:** Areas under the curve (AUC) of receiver operating characteristics curve (ROC) analysis and diagnostic testing

Test and cutoff values	AUC	Optimal	Sensitivity %	Specificity %	LR+	LR-	PPV %	NPV %
	(95% CI)	cutoff	(95% CI)	(95% CI)				
CRP	0.71 (0.67-0.73)	212 mg/L						
CRP >212 mg/L			51 (48-53)	80 (77-83)	2.55	0.62	89	35
PCT	0.70 (0.67-0.73)	2.45 mcg/L						
PCT >2.45 mcg/L			66 (63-68)	65 (60-71)	1.90	0.53	88	32
WBC	0.53 (0.5-0.55)	22.4 $\times 10^{9}/\hbox {L}$						
WBC>22.4$\times 10^{9}/\hbox {L}$			28 (26-30)	81 (77-84)	1.46	0.89	82	27
Body temp	0.59 (0.55-0.60)	$37.7^{\circ }\hbox {C}$						
Body temp> $38.0^{\circ }\hbox {C}$			25 (24-27)	85 (82-87)	1.64	0.88	83	27

The outcome was confirmed sepsis, with sepsis mimics as controls. Dichotomised CRP, PCT and WBC were based on an optimal cutoff (Youden’s index derived) from the ROC analysis, with high values considered positive tests. Confidence intervals (CIs) are left out for likelihood ratios (LR) and predictive values for the sake of readability

*CRP* C-reactive protein, *PCT* procalcitonin, *WBC* white blood cell count, *AUC* area under the curve, *CI* confidence interval, *LR* likelihood ratio, *PPV* positive predictive value, *NPV* negative predictive value, *Temp* temperature. *C* Celsius

**Fig. 4:**
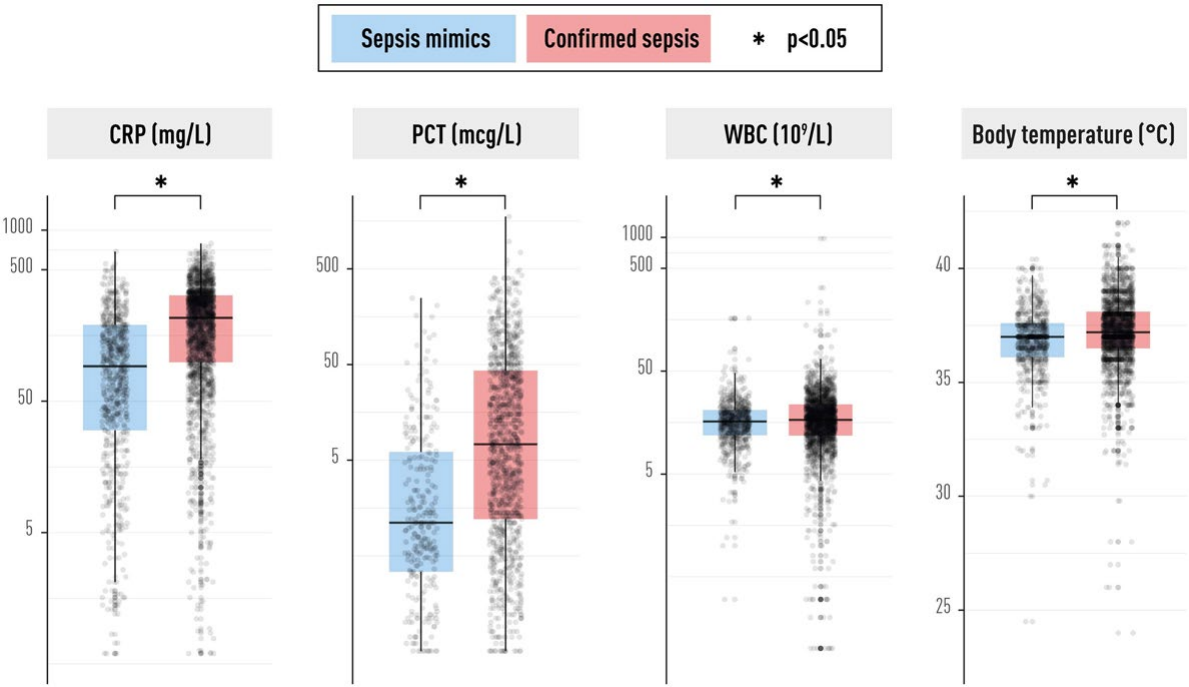
Boxplots of commonly used clinical variables. CRP, PCT and WBC are displayed on a log-10 scale because of non-normal distribution and outliers. Differences between sepsis mimics and confirmed sepsis were statistically significant according to a *p*-value <0.05 for all variables. *CRP* C-reactive protein, *PCT* procalcitonin, *WBC* white blood cell count

**Fig. 5:**
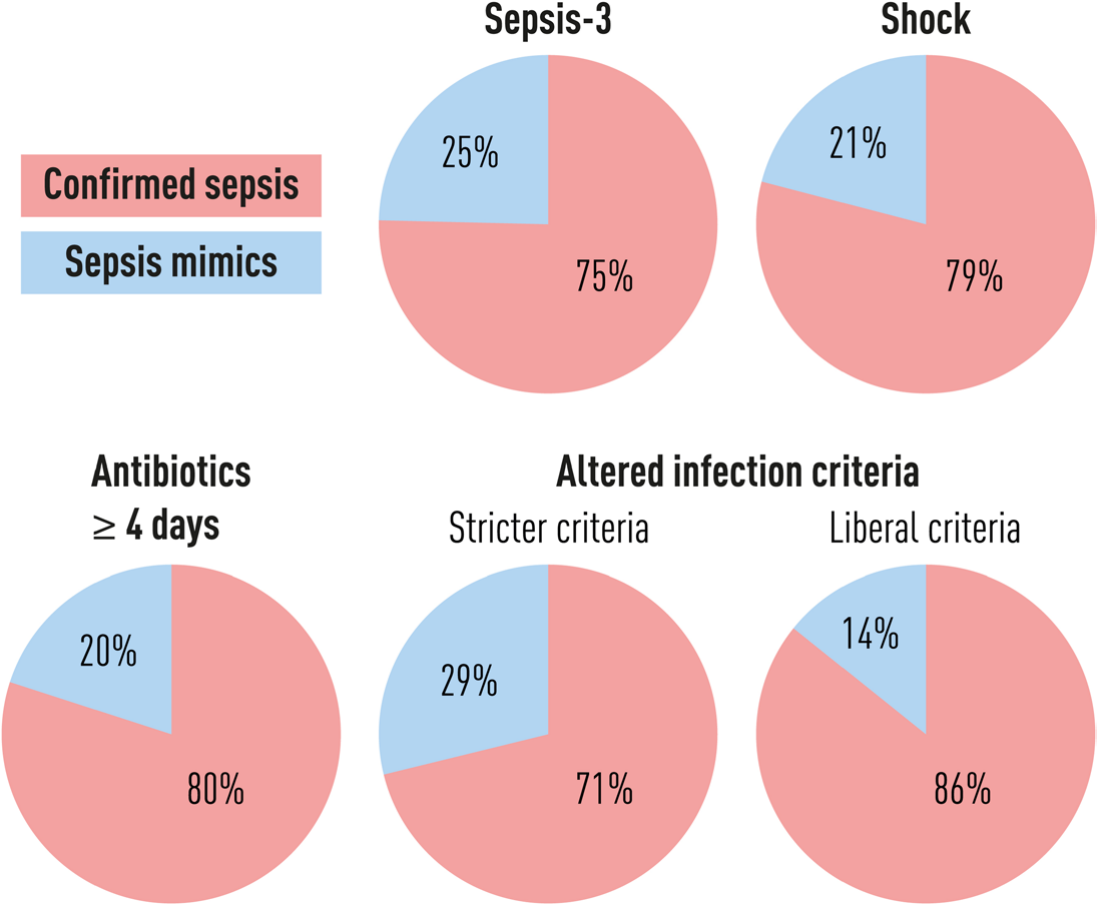
Sensitivity analyses of proportion of sepsis mimics with changing criteria for presumed sepsis and sepsis mimics. The main result of this study was that 25% of sepsis patients could be considered sepsis mimics when operational sepsis-3 criteria were used. When only sepsis patients with shock were included, the proportion of sepsis mimics fell to 21%. If only sepsis patients who received $\ge$4 days of antibiotics were included, the proportion of sepsis mimics fell to 20%. If the cutoff for infection was altered according to the Linder-Mellhammar criteria of infection (LMCI), the proportion of sepsis mimics was 29% with the stricter criteria (proven infection) and 14% with the more liberal criteria (at least possible infection). *LMCI* Linder-Mellhammar criteria of infection

## Supplementary Information

Below is the link to the electronic supplementary material.

Supplementary file1 Supplement 1 - method and results supplement.(PDF 160 kb)

Supplementary file2 Supplement 2 - diagnoses and diagnostic categories in sepsis mimics.(XLSX 23 kb)

Supplementary file3 Supplement 3 - variable list, data sources and proportions missing for variables in Table 1.(XLSX 13 kb)

## Data Availability

The datasets generated and analysed during the current study are not publicly available due to limitations in the ethical approval of the study and data management policies of Region Skåne. Still, they are available from the corresponding author on request.

## Abbreviations

AMI: Acute myocardial infarction
AUROC: Area under receiver operating curve
CA: Cardiac arrest
CDC: Center for disease control and prevention
COPD: Chronic obstructive pulmonary disorder
CRP: C-reactive protein
CRRT: Continuous renal replacement therapy
CT: Computer tomography
EMR: Electronic medical record
HT: Hypertensive
ICD-10: International statistical classification of diseases and related health problems version 10
ICH: Intracranial haemorrhage
ICU: Intensive care unit
IQR: Interquartile range
LMCI: The Linder-Mellhammar criteria of infection
LOS: Lenght of stay
LR: Likelihood ratio (+; positive, -; negative)
PF-ratio: PaO_2_/FiO_2_ (oxygenation index)
PCT: Procalcitonin
ROC: Receiver operating characteristics curve
SAPS-3: Simplified acute physiology score 3
SD: Standard deviation
SIR: Swedish intensive care registry
SOFA: Sequential organ failure assessment
STROBE: Strengthening the reporting of observational studies in epidemiology
WBC: White blood cell count

## Code availability

Available from the corresponding author on request.
