# Supplementary material for: Sepsis mimics among presumed sepsis patients at intensive care admission: a retrospective observational study
Source: Infection. 2024 Jan 27;52(3):1041–53. doi: 10.1007/s15010-023-02158-w (PMC11636973; doi:10.1007/s15010-023-02158-w)
Supplement: Supplementary file 1 — Supplementary file1 Supplement 1 - method and results supplement.(PDF 160 kb) [file 15010_2023_2158_MOESM1_ESM.pdf]

Supplement 1: Sepsis mimics among presumed sepsis patients at intensive care admission: a retrospective observational study

Contents

1 Methods supplement **1**
  1.1 **Definition of clinically relevant pathogens in microbiological samples** . 1
  1.2 **Sensitivity analysis two: proportion of sepsis mimics among patients with $\geq$ 4 days of antibiotic therapy** 2

2 Results supplement **3**
  2.1 ROC-analysis and diagnostic testing of the whole ICU population 3
  2.2 Sensitivity analyses 3

1 Methods supplement

1.1 Definition of clinically relevant pathogens in microbiological samples

Microbiological cultures were defined as clinically relevant if there was growth or detection of a pathogen from a culture taken within the stipulated time frame, regardless of the anatomical site of culturing. The following culture results were considered clinically irrelevant:

1. Yeast fungi from non-sterile anatomical sites (e.g. airways, lower urinary tract, skin lesions)

2. Potentially colonising bacteria of the upper or lower respiratory tract, if found in only one airway culture: Moraxella sp., coagulase-negative Staphylococci, viridans (alpha hemolytic) Streptococci

3. Potential skin contaminants found in only one blood culture: coagulase-negative Staphylococci, viridans (alpha hemolytic) Streptococci, micrococcus sp., Propionibacterium Acnes, Corynebacterium sp., Bacillus sp. [1]

4. Unspecific culture results (e.g. "gram-positive mixed flora", "vaginal flora", "skin flora", "anaerobic mixed flora") from non-sterile anatomical sites

5. Bacterial growth of Clostridium difficile, without detection of toxin [2]

6. Pneumococcus antigen tests from urinary samples

1.2 Sensitivity analysis two: proportion of sepsis mimics among patients with $\geq$ 4 days of antibiotic therapy

This sensitivity analysis was performed on patients from the two ICUs that registered administration of antibiotics in the electronic medical record (the other two ICUs used paper medical records or a separate electronic system, making data extraction difficult). ICU admissions were filtered according to the following criteria:
- no transfer between the four participating ICUs
- administration of $\geq$1 dose of antibiotic therapy (anatomical therapeutic chemical (ATC) code J01-J05) during the four first 24-hour periods following ICU admission.

2 Results supplement

2.1 ROC-analysis and diagnostic testing of the whole ICU population

S 1: **Areas under the curve (AUC) of ROC analysis and diagnostic testing.** The outcome was confirmed sepsis (n=2008), with all ICU non-sepsis (including sepsis mimics) as controls (n=6352). Dichotomised CRP, PCT and WBC were based on optimal cutoff from ROC analysis (Youden's index derived). *CRP; c-reactive protein. PCT; procalcitonin. WBC; white blood cell count. Temp; body temperature. AUC; area under the curve. CI; confidence interval. LR; likelihood ratio. PPV; positive predictive value. NPV; negative predictive value. C; Celsius.*

Test and cutoff	AUC (95% CI)	Optimal cutoff	Missing data %	Sensitivity % (95% CI)	Specificity % (95% CI)	LR+	LR-	PPV %	NPV %
CRP	0.77 (0.75-0.78)	124 mg/L	1.8%						
CRP>124 mg/L				69 (67-71)	69 (68-70)	2.24	0.44	42	87
PCT	0.71 (0.69-0.73)	3.45 mcg/L	69%						
PCT>3.45 mcg/L				61 (58-64)	70 (68-73)	2.05	0.55	61	70
WBC	0.60 (0.58-0.61)	17.5 $\times 10^9$/L	1.5%						
WBC>17.5$\times 10^9$/L				47 (45-49)	69 (68-70)	1.52	0.77	33	80
Temp	0.62 (0.60-0.63)	37.6°C	1.3%						
Temp>38.0°C				25 (24-27)	91 (91-92)	2.97	0.82	49	79

2.2 Sensitivity analyses

S 2: **Sensitivity analyses and hypothesis testing.** The table displays the number of sepsis mimics and presumed sepsis and the resulting proportion of sepsis mimics derived from each sensitivity analysis. The operational sepsis-3 criteria were used as the reference group in hypothesis testing.

Sensitivity analysis	Sepsis mimics, n	Presumed sepsis, n	Sepsis mimics(%)	P-value
Operational sepsis-3 criteria	*656*	*2664*	*25%*	
Shock	300	1436	21%	0.008
Antibiotics≥4days	149	741	20%	0.012
LMCI≥2	379	2664	14%	<0.001
LMCI≥4	768	2664	29%	<0.001

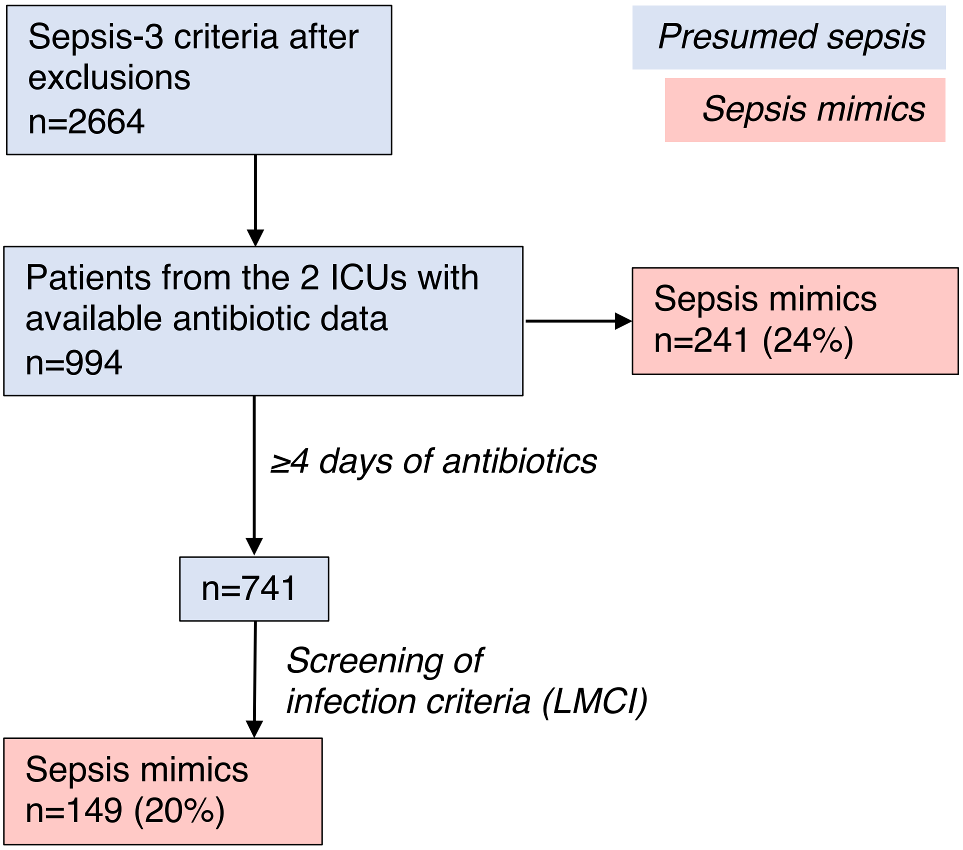

Figure 1: **Flow chart of sensitivity analysis four, including patients receiving ≥4 days of antibiotic therapy.** Since complete data on antibiotic therapy was only available for two of the four participating ICUs, this sensitivity analysis was only performed in patients from these two ICUs. The proportion of sepsis mimics fell slightly when a stricter proxy criteria for suspected infection was used, only including patients with longer antibiotic therapy. However, one-fifth of patients with presumed sepsis could still be considered sepsis mimics.

References

[1] Alcorn K, Meier F, Schifman R. Blood Culture Contamination: Q-tracks 2013. College of American Pathologists. 2013.

[2] Crobach M, Planche T, Eckert C, Barbut F, Terveer E, Dekkers O, et al. European Society of Clinical Microbiology and Infectious Diseases: update of the diagnostic guidance document for Clostridium difficile infection. Clinical microbiology and infection. 2016;22:S63-81.
